# Hydrodynamics of steady state phloem transport with radial leakage of solute

**DOI:** 10.3389/fpls.2013.00531

**Published:** 2013-12-26

**Authors:** Paulo Cabrita, Michael Thorpe, Gregor Huber

**Affiliations:** ^1^IBG-2: Plant Sciences, Forschungszentrum JülichJülich, Germany; ^2^Plant Sciences Division, Research School of Biology, Australian National UniversityCanberra, ACT, Australia

**Keywords:** phloem transport, fluid dynamics, navier-stokes equation, sieve tube, solute exchange

## Abstract

Long-distance phloem transport occurs under a pressure gradient generated by the osmotic exchange of water associated with solute exchange in source and sink regions. But these exchanges also occur along the pathway, and yet their physiological role has almost been ignored in mathematical models of phloem transport. Here we present a steady state model for transport phloem which allows solute leakage, based on the Navier-Stokes and convection-diffusion equations which describe fluid motion rigorously. Sieve tube membrane permeability *P*_*s*_ for passive solute exchange (and correspondingly, membrane reflection coefficient) influenced model results strongly, and had to lie in the bottom range of the values reported for plant cells for the results to be realistic. This smaller permeability reflects the efficient specialization of sieve tube elements, minimizing any diffusive solute loss favored by the large concentration difference across the sieve tube membrane. We also found there can be a specific reflection coefficient for which pressure profiles and sap velocities can both be similar to those predicted by the Hagen-Poiseuille equation for a completely impermeable tube.

## Introduction

Phloem transport denotes long-distance transport, mainly of assimilates arising from photosynthesis, and is the movement of a solution in a continuum of interconnected cells, sieve elements, within the phloem of the vascular tissues in plants. It is currently accepted that solutes enter and exit the sieve tubes at sources and sinks, water enters and exits osmotically, and the solution moves in these sieve tubes due to the consequent osmotically generated pressure gradient: the theory of Münch pressure flow.

However, there is also a considerable radial exchange of solutes between the sieve tubes and the adjacent cells along the long-distance pathway between source and sink regions, the so-called transport phloem (i.e., main veins, petioles, stems, and main roots) (Van Bel, 2003). This radial exchange has hardly been addressed in mechanistic modeling of phloem transport. In early work when there were vigorous discussions on the pathway mechanisms the flux was specified (e.g., Tyree et al., 1974; Goeschl et al., 1976), and more recently models of cambial growth in trees have included radial solute flux as a variable (among many processes) (De Schepper and Steppe, 2010; Hölttä et al., 2010). The radial flux can take both apoplastic and symplastic pathways, and either path can predominate according to plant species and conditions (Patrick and Offler, 1996). Here we consider the apoplastic path only, where exchange is believed to be a leak/retrieval system (Tegeder et al., 2012). The passive leak, diffusive but possibly facilitated, is driven by the high concentration difference between the sieve element/companion cell complex and its surrounding apoplast (Minchin et al., 1984; Minchin and Thorpe, 1987; Patrick, 1990; Van Bel, 1990; Carpaneto et al., 2005; Thorpe et al., 2005). The active retrieval is driven by sucrose/proton symport (Hafke et al., 2013). There is good direct evidence for the mechanism of retrieval in several species but, for unloading to be studied, no technique has yet given appropriate access. Passive unloading of photosynthates into the apoplast has been estimated at about 6% cm^−1^ in bean (*Phaseolus vulgaris*) (Minchin and Thorpe, 1987; Van Bel, 1990). This passive radial exchange of solutes is coupled with radial water flux. For example, leakage of radio-labeled photosynthates responds to changes in the apoplastic water potential [e.g., by perfusing the apoplast with sorbitol (Minchin et al., 1984); mannitol (Aloni et al., 1986; Cabrita, 2011) or polyethylene glycol (Cabrita, 2011)]. The leakage of other metabolites shows similar behavior (Aloni et al., 1986).

Given the current information about the complex process of solute exchange in the transport phloem, and in the belief that any realistic model for transport phloem should include such a process, we chose as a first step to consider only the passive leak, characterizing the sieve tube membrane by a membrane permeability *P*_*s*_ (in our case for sugars) and a reflection coefficient σ which describes the extent to which solute exchange is affected by the concentration difference across the membrane (Nobel, 2009). In all existing mathematical models of long-distance phloem transport based on the Münch pressure flow hypothesis [see Table 1 of Thompson and Holbrook (2003a)] the reflection coefficient was set to σ = 1, thus completely decoupling water exchange and solute exchange by assuming an ideally semipermeable membrane. The omission of radial solute exchange is not only a major gap in the description of phloem transport, but also makes the existing mathematical models suspect for interpreting the vast amount of experimental data that has been collected concerning phloem physiology.

Here we investigate the role of radial solute exchange in the transport phloem by studying a relatively simple system: steady state transport of a homogeneous solution (consisting of a single solute and water) in a tube surrounded by a bath of solution with predefined (but not necessarily constant) pressure and concentration. The simplicity of this setup allows us to highlight the effect of including radial solute exchange by comparing our findings with previous results from Thompson and Holbrook (2003b) and Phillips and Dungan (1993). In order to model the hydrodynamics of this system in a rigorous way we adapted a formalism introduced by Phillips and Dungan (1993), based on the Navier-Stokes and convection-diffusion equations. We extended this approach by introducing boundary conditions that specify radial exchange of solute across the sieve tube membrane. These exchange processes were described using the formalism of irreversible thermodynamics for transport across membranes (Kedem and Katchalsky, 1958). Our main focus is to study the importance for phloem transport of water and solute movement through the sieve tube membrane, and to evaluate the common practice in phloem flow modeling to assume semipermeable tubes.

## Materials and methods

### Model assumptions

**Figure 1:**
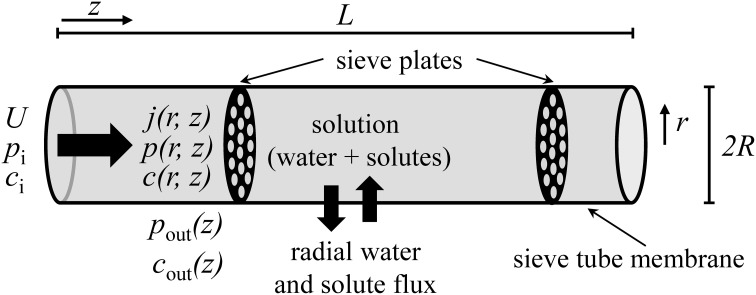
**Sieve tube model**. Solution enters the sieve tube with velocity *U*, pressure *p*_i_ and concentration *c*_i_. There is radial exchange of water and solute across the sieve tube membrane along the pathway. The sieve tube is surrounded by a medium (“apoplast”) with pressure *p*_out_(*z*) and concentration *c*_out_(*z*). *R* and *L* are sieve tube radius and length, respectively. *r* and *z* are the directions of radial and axial flow, respectively.

We propose a hydrodynamic model of phloem transport, according to the Münch pressure flow hypothesis, with allowance for water and solute exchange along the pathway corresponding to the transport phloem (Van Bel, 2003) (Figure 1). The following assumptions are made:

1. The sieve tube is considered as a right circular cylinder of length *L* and radius *R*, such that *R* « *L*, limited by a porous membrane through which water and solutes fluxes occur. We use cylindrical coordinates where the axis along the pathway is denoted by *z* = [0, *L*], the radial coordinate is *r* = [0, *R*] and φ = [0, 2π ] is the azimuth. The tube is surrounded by unspecified tissue (for simplicity called apoplast in the following).
2. The flow is axisymmetrical, i.e., flow depends on axis coordinate *z* and radial coordinate *r*, but not on azimuth φ.
3. Sieve tube sap is regarded as a homogeneous solution of a single solute with concentration *c*(*z, r*) in water and assumed to be an incompressible Newtonian fluid of constant density, ρ. Sieve tube sap viscosity, μ, is constant, i.e., independent of solute concentration.
4. End effects caused by the entry and exit of sap in the sieve tube are negligible.
5. The system is at steady state, i.e., time independent.
6. The sap enters the sieve tube with an average speed *U*, with an average solute concentration *c*_i_ and at average turgor pressure *p*_i_.
7. The membrane hydraulic conductivity, *L*_p_, is constant. Solute exchange across the membrane is regarded as a passive process in which there is: (i) diffusion of solutes that is linear with the concentration difference between the sieve tube and the surrounding apoplast, and dependent on the sieve tube membrane solute permeability, *P*_*s*_; (ii) convection of solutes dragged by water through the membrane.
8. Apoplast solute concentration, *c*_out_(*z*), and hydrostatic pressure *p*_out_(*z*) are not affected by solute or water exchange across the sieve tube membrane. These functions have to be provided as boundary conditions.
9. There is a constant water potential gradient $\frac{d\Psi_{\text{out}}}{dz}$ surrounding the sieve tube.
10. Diffusion of solute within sieve tube sap is isotropic and obeys Fick's law of diffusion with a constant diffusion coefficient *D*, solute-specific.
11. There is no slip at the sieve tube membrane.
12. There are no chemical reactions.
13. For simplicity, we assume osmotic pressure to be given by van't Hoff equation for dilute solutions: Π (*z*) = *R*_*g*_*Tc*(*z*), where *R*_*g*_ is the universal gas constant and *T* the absolute temperature.
14. Sieve plates are transverse and spaced at regular intervals (Figure 1). The effect of sieve plates on the overall sieve tube conductance is described by an impedance factor β which is related to sieve tube element structure and given by Thompson and Holbrook (2003a):
  (1) $$\beta =\frac{8\alpha r_{\text{p}}^{2}l}{\left(8l_{\text{p}}+3\pi r_{\text{p}}\right)R^{2}+8\alpha r_{\text{p}}^{2}\left(l-l_{\text{p}}\right)},\;r_{p}<R$$

where α is the fraction of sieve plate area composed of sieve pore area; *l* is the length of sieve tube elements in between sieve plates; *r*_*p*_ and *l*_*p*_ define the sieve plate pore dimensions: radius and length respectively (Thompson and Holbrook, 2003a). As α = 1, *l* > *l*_*p*_ and *R* > *r*_*p*_ we have always β < 1. For the special case of no sieve plates the impedance factor is set to β = 1.

### Fundamental equations

As in Phillips and Dungan (1993) the calculation starts from the steady state Navier-Stokes equation for an incompressible Newtonian fluid which, using the assumption of axisymmetrical flow, can be written in cylindrical coordinates as (Truskey et al., 2009)

(2) $$\rho \left(u_{r}\frac{\partial u_{r}}{\partial r}+u_{z}\frac{\partial u_{r}}{\partial z}\right)=-\frac{\partial p}{\partial r}+\mu^{*}\left[\frac{\partial }{\partial r}\left(\frac{1}{r}\frac{\partial \left(ru_{r}\right)}{\partial r}\right)+\frac{\partial^{2}u_{r}}{\partial z^{2}}\right]\;$$

(3) $$\rho \left(u_{r}\frac{\partial u_{z}}{\partial r}+u_{z}\frac{\partial u_{z}}{\partial z}\right)=-\frac{\partial p}{\partial z}+\mu^{*}\left[\frac{1}{r}\frac{\partial }{\partial r}\left(r\frac{\partial u_{z}}{\partial r}\right)+\frac{\partial^{2}u_{z}}{\partial z^{2}}\right]$$

where pressure *p* and velocities *u*_*r*_ and *u*_*z*_ are functions of axial coordinate *z* and radial coordinate *r*. In most cases we omit these dependences in our notation for simplicity. The effect of sieve plates is described introducing an effective viscosity μ^*^ = μ/β, thus having a more viscous fluid as β = 1 [see Equation (1)]. This approach treats the sieve tube as a conduit of essentially uniform resistivity, a close approximation to reality in view of the distribution of the sieve plates within sieve tubes (Weir, 1981; Henton et al., 2002). Pressure *p* includes the gravitational effect, i.e., *p* = *p*′ + ρ*gh*; where *p'* is termed the hydrodynamic pressure inside the sieve tube, *g* the local acceleration of gravity and *h* is the vertical coordinate above a standard reference plane. Note that in contrast to Phillips and Dungan (1993) we use the Navier-Stokes equation including inertial forces [left hand side of Equations (2) and (3), respectively] to keep the formulation as general as possible. The actual impact of inertial forces is discussed in the Results section. The continuity equation for incompressible fluids in cylindrical coordinates is (Truskey et al., 2009)

(4) $$\frac{1}{r}\frac{\partial \left(ru_{r}\right)}{\partial r}+\frac{\partial u_{z}}{\partial z}=0$$

and the convection-diffusion equation for dilute solutions in steady state is (Truskey et al., 2009)

(5) $$u_{r}\frac{\partial c}{\partial r}+u_{z}\frac{\partial c}{\partial z}=D\left[\frac{1}{r}\frac{\partial }{\partial r}\left(r\frac{\partial c}{\partial r}\right)+\frac{\partial^{2}c}{\partial z^{2}}\right]$$

where concentration *c* is a function of coordinates *z* and *r* in the same way as pressure and velocities. The axial and radial fluxes of solute inside the sieve tube are defined as sum of convective and diffusive components

(6) $$j_{z}^{\text{s}}=u_{z}c-D\frac{\partial c}{\partial z}$$

(7) $$j_{r}^{\text{s}}=u_{r}c-D\frac{\partial c}{\partial r}$$

### Boundary conditions

To allow for solute and water exchanges across the sieve tube membrane, the following boundary conditions are used: radial flux of solution through the membrane (at *r* = *R*) is given by Starling's equation (Nobel, 2009):

(8) $$u_{r}(R,z)=L_{\text{P}}\left\{p\left(R,z\right)-p_{\text{out}}\left(z\right)-\sigma R_{\text{g}}T\left[c\left(R,z\right)-c_{\text{out}}\left(z\right)\right]\right\}$$

where the van't Hoff expression of osmotic pressure for dilute solution has been inserted. The reflection coefficient of the sieve tube membrane σ assumes a value between 0 (totally permeable) and 1 (semipermeable). Pressure, *p*_out_, and solute concentration *c*_out_, outside the tube do not to depend on *r* and are assumed to vary linearly with distance:

(9) $$p_{\text{out}}(z)=p_{\text{out}}(0)+\frac{dp_{\text{out}}}{dz}z$$

and

(10) $$c_{\text{out}}(z)=c_{\text{out}}(0)+\frac{dc_{\text{out}}}{dz}z$$

Radial passive flux of solutes through the membrane on the one hand is given by Equation (7) for *r* = *R*, on the other hand it can be defined as the sum of convective flux (solute dragged by solvent) which is linear with the average solute concentration in the membrane, *c*(*z*), plus diffusive flux across the membrane that is described by the sieve tube membrane solute permeability, *P*_*s*_, and the concentration difference across the membrane (Kedem and Katchalsky, 1958; Benedek and Villars, 2000).

(11) $$\begin{array}{l} j_{r}^{\text{s}}(R,z)=u_{r}(R,z)c(R,z)-D\frac{\partial c}{\partial r}(R,z)\\ \;=(1-\sigma )u_{r}(R,z)\bar{c}(z)+P_{s}\left[c\left(R,z\right)-c_{\text{out}}\left(z\right)\right] \end{array}$$

The concentration of solute, *c*(*z*), within the sieve tube membrane is given by $\bar{c}(z)=\frac{c(R,z)+c_{\text{out}}(z)}{2}$. The no-slip condition implies that

(12) $$u_{z}(R,z)=0$$

which means that the fluid velocity will be in the radial direction only, at the boundary.

Symmetry at the center of the tube implies that radial velocity as well as radial derivatives of axial velocity and concentration are zero at *r* = 0: *u*_*r*_ (0, *z*) = 0, $\frac{\partial u_{z}}{\partial r}(0,z)=0$ and $\frac{\partial c}{\partial r}\left(0,z\right)=0$. According to our model assumptions, flow is already fully developed at the beginning of the transport phloem region considered in our model; loading of solutes and water has occurred in the source region, not being part of our model upstream. Therefore, the boundary conditions at the inlet of the tube *z* = 0 are 〈*u*_*z*_(0)〉 = *U*, 〈*c*(0)〉 = *c*_i_ and 〈*p*(0)〉 = *p*_i_ where the bracket denotes the average over the cross section $\langle c(z)\rangle =\frac{2}{R^{2}}\int_{0}^{R}c(r,z)rdr$.

Note that the hydrodynamic equations (2) to (4) describing the solution movement inside the sieve tube do not depend on the solute concentration explicitly, and we take viscosity to be independent of concentration. The flow depends on concentration through the boundary condition Equation (8) only. Thus, we can treat independently the hydrodynamics problem that describes how pressure and velocity change, and the solute transport equation (5) which is solved once the velocity field is determined.

### Dimensional analysis

As with many problems in fluid dynamics and especially when applying the Navier-Stokes equation, the problem becomes more simple to solve using dimensional analysis (Regirer, 1960; Kundu and Cohen, 2008). With this method, it is possible to predict physical parameters that influence the sap flow and determine the relationships between several variables (pressure, velocity and concentration) when an exact functional relationship is unknown. This is not possible with a direct numerical solution of the governing equations (2) to (5). Following Regirer (1960), Phillips and Dungan (1993) and Thompson and Holbrook (2003b), we use system geometry and boundary conditions as scales to define dimensionless variables in the following way:

(13) $$\hat{r}=\frac{r}{R},\hat{z}=\frac{z}{L},{\hat{u}}_{z}=\frac{u_{z}}{U}\text{ and }\hat{c}=\frac{c}{c_{\text{i}}}$$

Inserting these definitions into the governing equations, the corresponding scaling of the remaining variables and parameters [unique except for numerical prefactors which were chosen according to Thompson and Holbrook (2003a)] becomes

$$\begin{array}{l} \hat{p}=p_{\hat{\text{i}}}\frac{R^{2}}{8LU\mu^{*}},{\hat{u}}_{r}=u_{r}\frac{L}{RU},{\hat{j}}_{z}^{s}=\frac{j_{z}^{s}}{Uc_{i}},{\hat{j}}_{r}^{s}=j_{r}^{s}\frac{L}{RUc_{i}},\\ {\hat{L}}_{\text{p}}=L_{\text{p}}\frac{16L^{2}\mu^{*}}{R^{3}},{\hat{P}}_{\text{s}}=P_{\text{s}}\frac{2L}{RU} \end{array}$$

Substituting the dimensionless variables into the governing equations, the dimensionless Navier-Stokes Equations (2) and (3) become:

(14) $$\text{Re }\varepsilon^{3}\left({\hat{u}}_{r}\frac{\partial {\hat{u}}_{r}}{\partial \hat{r}}+{\hat{u}}_{z}\frac{\partial {\hat{u}}_{r}}{\partial \hat{z}}\right)=-8\frac{\partial \hat{p}}{\partial \hat{r}}+\varepsilon^{2}\left[\frac{\partial }{\partial \hat{r}}\left(\frac{1}{\hat{r}}\frac{\partial \left(\hat{r}{\hat{u}}_{r}\right)}{\partial \hat{r}}\right)+\varepsilon^{2}\frac{\partial^{2}{\hat{u}}_{r}}{\partial {\hat{z}}^{2}}\right]$$

(15) $$\text{Re}\varepsilon \left({\hat{u}}_{r}\frac{\partial {\hat{u}}_{z}}{\partial \hat{r}}+{\hat{u}}_{z}\frac{\partial {\hat{u}}_{z}}{\partial \hat{z}}\right)=-8\frac{\partial \hat{p}}{\partial \hat{z}}+\frac{1}{\hat{r}}\frac{\partial }{\partial \hat{r}}\left(\hat{r}\frac{\partial {\hat{u}}_{z}}{\partial \hat{r}}\right)+\varepsilon^{2}\frac{\partial^{2}{\hat{u}}_{z}}{\partial {\hat{z}}^{2}}$$

Here ε = *R*/*L* is an abbreviation for the aspect ratio and Re = ρ*RU*/μ^*^ is the Reynolds number, the ratio of inertial to viscous forces. The continuity equation (4) now reads

(16) $$\frac{1}{\hat{r}}\frac{\partial \left(\hat{r}{\hat{u}}_{r}\right)}{\partial \hat{r}}+\frac{\partial {\hat{u}}_{z}}{\partial \hat{z}}=0$$

The convection diffusion equation (5) becomes

(17) $$\varepsilon \left({\hat{u}}_{r}\frac{\partial \hat{c}}{\partial \hat{r}}+{\hat{u}}_{z}\frac{\partial \hat{c}}{\partial \hat{z}}\right)=\frac{1}{{\text{Pe}}_{r}}\left[\frac{1}{\hat{r}}\frac{\partial }{\partial \hat{r}}\left(\hat{r}\frac{\partial \hat{c}}{\partial \hat{r}}\right)+\varepsilon^{2}\frac{\partial^{2}\hat{c}}{\partial {\hat{z}}^{2}}\right]$$

where Pe_*r*_ = *RU/D* is the Péclet number for radial flow. The components of the sieve tube solute flux are given by:

(18) $${\hat{j}}_{r}^{\text{s}}=-\frac{1}{{\text{Pe}}_{r}}\frac{\partial \hat{c}}{\partial \hat{r}}+\varepsilon {\hat{u}}_{r}\hat{c}$$

(19) $${\hat{j}}_{z}^{\text{s}}=-\frac{1}{{\text{Pe}}_{z}}\frac{\partial \hat{c}}{\partial \hat{z}}+{\hat{u}}_{z}\hat{c}$$

where Pe_*z*_ = *LU/D* = Pe_*r*_/ε is the Péclet number for axial flow. The Péclet number for any direction gives the ratio of the rate of convection of the solute in that direction by the flow of sap, to the corresponding rate of diffusion of that solute driven by a concentration gradient.

In dimensionless notation, the boundary conditions Equations (8), (11) and (12) at the sieve tube membrane are

(20) $${\hat{u}}_{r}(1,\hat{z})=\frac{{\hat{L}}_{\text{P}}}{2}\left\{\hat{p}\left(1,\hat{z}\right)-{\hat{p}}_{\text{out}}\left(\hat{z}\right)-\sigma \hat{H}\left[\hat{c}\left(1,\hat{z}\right)-{\hat{c}}_{\text{out}}\left(\hat{z}\right)\right]\right\}$$

(21) $$\begin{array}{l} \frac{1}{{\text{Pe}}_{r}}\frac{\partial \hat{c}}{\partial \hat{r}}\left(1,\hat{z}\right)=\varepsilon {\hat{u}}_{r}\left(1,\hat{z}\right)\left[\hat{c}\left(1,\hat{z}\right)-\left(1-\sigma \right)\hat{\bar{c}}\left(\hat{z}\right)\right]-\varepsilon \frac{{\hat{P}}_{s}}{2}[\hat{c}\left(1,\hat{z}\right)\\ \;-{\hat{c}}_{\text{out}}\left(\hat{z}\right)] \end{array}$$

(22) $${\hat{u}}_{z}\left(1,\hat{z}\right)=0$$

The dimensionless parameter $\hat{H}=\frac{R_{g}Tc_{\text{i}}R^{2}}{8LU\mu^{*}}$, defined by $\hat{\Pi }=\hat{H}\hat{c}$, gives the ratio of osmotic forces to viscous forces for a flow with a characteristic velocity *U* (Phillips and Dungan, 1993).

The boundary conditions at the center of the tube become *û*_*r*_ (0, *ẑ*) = 0, $\frac{\partial {\hat{u}}_{z}}{\partial \hat{r}}\left(0,\hat{z}\right)=0$ and $\frac{\partial \hat{c}}{\partial \hat{r}}\left(0,\hat{z}\right)=0$. Boundary conditions at *ẑ* = 0 are 〈*û*_*z*_〉 = 1, 〈*ĉ*〉 = 1 and 〈$\hat{p}$〉 = $\hat{p}$_i_. Thus after non-dimensionalization the parameters governing the model behavior are ${\hat{L}}_{\text{p}}$, *Ĥ*, $\hat{P}$_s_, σ, ε, Re, Pe_*r*_ together with boundary condition values $\hat{p}$_i_, $\hat{p}$_out_ (*ẑ*) and *ĉ*_out_ (*ẑ*).

### Series expansion

Proceeding in the same way as Phillips and Dungan (1993) we expand the state variables, *û*_*r*_, *û*_*z*_, *ĉ* and $\hat{p}$ as power series of the small dimensionless aspect ratio $\varepsilon :{\hat{u}}_{r}=\sum_{j=0}^{\infty }\varepsilon^{j}{\hat{u}}_{rj},{\hat{u}}_{z}=\sum_{j=0}^{\infty }\varepsilon^{j}{\hat{u}}_{zj},\hat{c}=\sum_{j=0}^{\infty }\varepsilon^{j}{\hat{c}}_{j}$ and $\hat{p}=\sum_{j=0}^{\infty }\varepsilon^{j}{\hat{p}}_{j}$. The accuracy of the expansions, i.e., the number of terms to include, will depend on the value of ε: the smaller ε, the more significant are the first terms compared to higher order terms. In the case of phloem transport, ε is smaller than 10^−3^ for typical sieve tube dimensions (Esau, 1969). For this reason we will only consider the first two terms (zeroth and first order) of the expansion to describe flow in a sieve tube. Higher order terms might be of interest when studying effects on a smaller length scale (e.g., single sieve tube elements).

Collecting terms of the appropriate order (ε^0^ and up to ε^1^, respectively), using respective boundary conditions and inserting zeroth order results into the calculation of first order leads to the following expressions: axial velocity can be calculated from Equations (15) and (22) as

(23) $${\hat{u}}_{z0}\left(\hat{r},\hat{z}\right)\text{​}=\text{​}2\frac{\partial {\hat{p}}_{0}}{\partial \hat{z}}\left({\hat{r}}^{2}-1\right)$$

(24) $${\hat{u}}_{z1}\left(\hat{r},\hat{z}\right)\text{​}=\text{​}2\frac{\partial {\hat{p}}_{1}}{\partial \hat{z}}\left({\hat{r}}^{2}\text{​}-\text{​}1\right)+\frac{\text{Re}}{36}\frac{\partial^{2}{\hat{p}}_{0}}{\partial {\hat{z}}^{2}}\frac{\partial {\hat{p}}_{0}}{\partial \hat{z}}\left(2{\hat{r}}^{6}\text{​}-\text{​}9{\hat{r}}^{4}\text{​}+\text{​}36{\hat{r}}^{2}\text{​}-\text{​}29\right)\;$$

where pressures $\hat{p}$_0_ and $\hat{p}$_1_ are independent of radial coordinate *r* as a result of Equation (14). Using Equations (23) and (24), radial velocity follows from Equation (16) as

(25) $${\hat{u}}_{r0}\left(\hat{r},\hat{z}\right)=-\frac{1}{2}\frac{\partial^{2}{\hat{p}}_{0}}{\partial {\hat{z}}^{2}}\left({\hat{r}}^{3}-2\hat{r}\right)$$

(26) $$\begin{array}{l} {\hat{u}}_{r1}\left(\hat{r},\hat{z}\right)=-\frac{1}{2}\frac{\partial^{2}{\hat{p}}_{1}}{\partial {\hat{z}}^{2}}\left({\hat{r}}^{3}-2\hat{r}\right)-\frac{\text{Re}}{144}\frac{\partial }{\partial \hat{z}}\left(\frac{\partial^{2}{\hat{p}}_{0}}{\partial {\hat{z}}^{2}}\frac{\partial {\hat{p}}_{0}}{\partial \hat{z}}\right)\\ \;\left({\hat{r}}^{7}-6{\hat{r}}^{5}+36{\hat{r}}^{3}-58\hat{r}\right) \end{array}$$

Inserting Equation (20) into Equations (25) and (26) the relation of pressure and concentration becomes

(27) $$\begin{array}{l} \frac{\partial^{2}{\hat{p}}_{0}}{\partial {\hat{z}}^{2}}={\hat{L}}_{\text{P}}\left\{{\hat{p}}_{0}\left(\hat{z}\right)-{\hat{p}}_{\text{out}}\left(\hat{z}\right)-\sigma \hat{H}\left[{\hat{c}}_{0}\left(1,\hat{z}\right)-{\hat{c}}_{\text{out}}\left(\hat{z}\right)\right]\right\}\\ \;=:{\hat{u}}_{\text{lat 0}}\left(\hat{z}\right) \end{array}$$

(28) $$\frac{\partial^{2}{\hat{p}}_{1}}{\partial {\hat{z}}^{2}}\text{​}=\text{​}{\hat{L}}_{\text{P}}\left\{{\hat{p}}_{1}\left(\hat{z}\right)\text{​}-\text{​}\sigma \hat{H}{\hat{c}}_{1}\left(1,\hat{z}\right)\right\}\text{​}-\text{​}\frac{3\text{Re}}{8}\frac{\partial }{\partial \hat{z}}\left(\frac{\partial^{2}{\hat{p}}_{0}}{\partial {\hat{z}}^{2}}\frac{\partial {\hat{p}}_{0}}{\partial \hat{z}}\right)\;$$

The average of zeroth order axial velocity over the cross section is $\langle {\hat{u}}_{z0}\rangle =-\frac{\partial {\hat{p}}_{0}}{\partial \hat{z}}$. Thus in our model the pressure gradient at any distance from the origin is linear with the average axial velocity, but dependent both explicitly and implicitly on the radial flux of water at that location, given by the zeroth order function *û*_lat 0_ (*ẑ*), see Equation (27).

Evaluating the convection diffusion equation (17) for ε = 0 together with the boundary condition, we conclude *ĉ*_0_ (*ẑ*) to be independent of radial coordinate: $\frac{\partial {\hat{c}}_{0}}{\partial \hat{r}}=0$. Using this result and Equation (23) the first order expression of Equation (17) becomes

(29) $$\frac{\partial {\hat{c}}_{1}}{\partial \hat{r}}=\frac{{\text{Pe}}_{r}}{2}\frac{\partial {\hat{p}}_{0}}{\partial \hat{z}}\frac{\partial {\hat{c}}_{0}}{\partial \hat{z}}\left({\hat{r}}^{3}-2\hat{r}\right)$$

The first order expression of the boundary condition Equation (21) is

(30) $$\begin{array}{l} \frac{1}{{\text{Pe}}_{r}}\frac{\partial {\hat{c}}_{1}}{\partial \hat{r}}\left(1,\hat{z}\right)={\hat{u}}_{r0}\left(1,\hat{z}\right)\left[{\hat{c}}_{0}\left(1,\hat{z}\right)-\left(1-\sigma \right){\hat{\bar{c}}}_{0}\left(\hat{z}\right)\right]\\ \;-\;\frac{{\hat{P}}_{s}}{2}\left[{\hat{c}}_{0}\left(1,\hat{z}\right)-{\hat{c}}_{\text{out}}\left(\hat{z}\right)\right] \end{array}$$

Combining Equations (29) and (30) and inserting Equations (25) and (27) leads to the following zeroth order equation for concentration

(31) $$\frac{\partial {\hat{p}}_{0}}{\partial \hat{z}}\frac{\partial {\hat{c}}_{0}}{\partial \hat{z}}=-{\hat{u}}_{\text{lat 0}}\left({\hat{c}}_{0}-(1-\sigma ){\hat{\bar{c}}}_{0}\right)+{\hat{P}}_{s}\left({\hat{c}}_{0}-{\hat{c}}_{\text{out}}\right)$$

The final first order expression for concentration follows from Equation (29) using the boundary conditions 〈*ĉ*_1_ (*z* = 0)〉 = 0 and 〈*û*_*z*0_ (*z* = 0)〉 = −∂$\hat{p}$_0_(*z* = 0)/∂*ẑ* = 1

(32) $${\hat{c}}_{1}\left(\hat{r},\hat{z}\right)=\frac{{\text{Pe}}_{r}}{8}\frac{\partial {\hat{p}}_{0}}{\partial \hat{z}}\frac{\partial {\hat{c}}_{0}}{\partial \hat{z}}\left({\hat{r}}^{4}-4{\hat{r}}^{2}\right)-\frac{5{\text{Pe}}_{r}}{24}\frac{\partial {\hat{c}}_{0}(z=0)}{\partial \hat{z}}$$

As the velocity profile depends on pressure only [Equations (23) to (26)] and first order concentration follows from zeroth order expressions [Equation (32)] we need to know *ĉ*_0_, $\hat{p}$_0_, and $\hat{p}$_1_ only in order to obtain the pressure and velocity profiles and thus describe phloem flow up to first order of the expansion in aspect ratio ε. These variables are obtained by solving the system of coupled differential equations (27), (28) and (31).

For a semipermeable membrane, i.e., σ = 1 and $\hat{P}$_*s*_ = 0, the zeroth order equations (23), (25), (27) and (31) are identical to findings of Phillips and Dungan (1993) except for different numerical prefactors stemming from slightly different definitions of the dimensionless quantities. Also in this case Equations (27) and (31) are equivalent to the governing equations of Thompson and Holbrook (2003b), the only difference being in the boundary conditions. In this light it is not surprising that the zeroth order results Equations (27) and (31) for a permeable membrane can also be obtained using an approach similar to Thompson and Holbrook (2003a), based on conservation equations and a local application of the Hagen-Poiseuille equation together with appropriate boundary conditions, see Appendix B.

### Numerical analysis

As there is no analytical solution for the system of differential Equations (27), (28) and (29), they were transformed into a first order system of differential equations [Appendix C, Equations (C.3)–(C.7)] and solved with MATLAB (R2010b, MathWorks, Inc.) ode15s differential equations solver routine. The calculated zeroth order of concentration, *ĉ*_0_, turgor pressure, $\hat{p}$_0_, the first order of turgor pressure, $\hat{p}$_1_, and their respective derivatives were then used to determine the average first order concentration 〈*ĉ*_1_〉, Equation (C.8). Concentration profile and sieve tube turgor pressure were determined as sum of zeroth and first order. From these profiles, average over cross section of axial velocity Equation (C.9), radial flow at sieve tube membrane Equation (C.10), axial solute flux Equation (19) and radial solute flux Equation (18) were determined.

### Parameter values

Unless otherwise specified, the parameter values of Table 1 were used (the basis for these values is given in Appendix A). The values of the non-dimensional model parameters related to the parameter values of Table 1 are shown in Table 2.

**Table 1:** **Values of the physical parameters chosen to represent the phloem**.

**Parameter**	**S.I. Unit**	**Value**
Apoplast pressure, *p*_out_(0)	MPa	0.1
Apoplast solute concentration, *c*_out_(0)	mol m^−3^	60
Apoplast osmotic pressure gradient, $\frac{d\Pi_{\text{out}}}{dz}$	MPa m^−1^	0.01
Apoplast pressure gradient, $\frac{dp_{\text{out}}}{dz}$	MPa m^−1^	0.04
Fraction of sieve plate area occupied by pores, α	–	0.5^a^
Flow speed at the origin, *U*	m s^−1^	1.7 × 10^−4^
Turgor pressure at the origin, *p*_i_	MPa	1.0
Sieve tube solute concentration at the origin, *c*_i_	mol m^−3^	600
Pathway length, *L*	m	0.5
Sap viscosity, μ	Pa s	0.0015
Sieve plate pore radius, *r*_*p*_	μm	0.23^a^
Sieve plate length, *l*_*p*_	μm	0.5^a^
Sieve plate impedance factor, β	—	0.079^a^
Sieve tube element length, *l*	μm	250^a^
Sieve tube membrane hydraulic conductivity, *L*_*p*_	m s^−1^ Pa^−1^	5 × 10^−15^
Sieve tube radius, *R*	μm	10
Sieve tube solute permeability, *P*_*s*_	m s^−1^	1.83 × 10^−9^ (1 − σ) ^d^
Sucrose diffusion coefficient, *D*	m^2^ s^−1^	4.6 × 10^−10^^b^
Sucrose specific volume, *V*_*S*_	m^3^ mol^−1^	2.155 × 10^−4^^c^
Temperature, *T*	°C	22
Universal gas constant, *R*_*g*_	J K^−1^ mol^−1^	8.314
Water density, *ρ*_*w*_	kg m^−3^	998

a *Thompson and Holbrook (2003a)*.

b *Phillips and Dungan (1993)*.

c *Eszterle (1993)*.

d *Appendix A*.

**Table 2:** **Values of non-dimensional physical parameters resulting from data of Table 1**.

**Parameter**	**Value**
*ĉ*_out_ (*ẑ*)	0.1 + 0.00679*ẑ*
ε	2 × 10^−5^
${\hat{L}}_{\text{p}}$	0.3797
*Ĥ*	11.405
$\hat{p}$_i_	7.746
$\hat{P}$_s_	1.038 (1–σ)
Pe_*r*_	3.696
$\hat{p}$_out_ (*ẑ*)	0.7746 + 0.3098*ẑ*
Re	8.94 × 10^−5^
σ	0 to 1

## Results

### Permeable membrane—the effect of radial solute exchange

**Figure 2:**
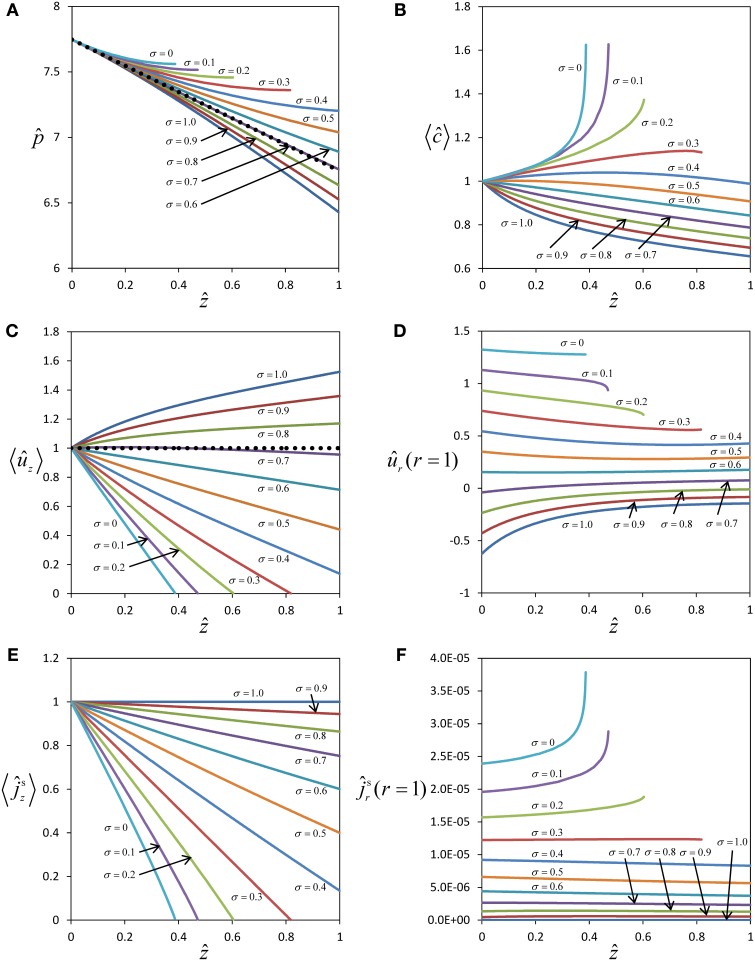
**Effect of solute permeability on the flow within a sieve tube limited by a permeable membrane**. Pressure **(A)**, average concentration **(B)**, average axial velocity **(C)**, radial velocity at sieve tube membrane **(D)**, axial **(E)** and radial **(F)** solute fluxes with position *ẑ* for different reflection coefficients (and therefore solute permeability). Pressure and axial velocity profiles for the Poiseuille flow regime are also shown (••••••) in **(A)** and **(C)** respectively.

Figure 2 shows profiles of pressure, concentration and flow for different values of the reflection coefficient σ. In the case of a semipermeable membrane (σ = 1), concentration and pressure differences across the sieve tube membrane (*c* and *p* are higher than *c*_out_ and *p*_out_) create a water potential difference, ΔΨ, across the sieve tube membrane that always draws water into the sieve tube (*û*_*r*_ < 0, Figure 2D), causing a pronounced non-linearity of pressure in the direction of flow (Figure 2A). In the case of a membrane that is permeable to solutes as well as water (σ < 1), the water influx depends on the value of the reflection coefficient σ and can even be zero (Figure 2D), leading to a pressure profile almost identical to Poiseuille flow (Figure 2A). Boundary condition Equation (20) shows that a water influx (*û*_*r*_ < 0) will occur as long as:

(33) $$\sigma >\frac{\hat{p}\left(1,z\right)-{\hat{p}}_{\text{out}}\left(z\right)}{\hat{H}\left[\hat{c}\left(1,z\right)-{\hat{c}}_{\text{out}}\left(z\right)\right]}$$

For typical physiological conditions (Table 1), i.e., for the order of magnitude of pressure and concentration expected for sieve tubes, water influx into the sieve tube occurs only if σ > 0.7 (Figure 2D). If the permeability of the membrane, *P*_*s*_, increases (with σ decreasing from σ = 1 to σ = 0.7; see Appendix A for the relationship of *P*_*s*_ and σ), the absolute value of the pressure gradient in the direction of flow becomes smaller with decreasing axial velocity (Figure 2C) as $\langle {\hat{u}}_{z}\rangle \approx -\frac{\partial \hat{p}}{\partial \hat{z}}$, see Equation (C.9). This trend arises from two factors that decrease the sieve tube solute concentration along the axis: first, the dilution created by water influx, dependent on the water potential difference across the sieve tube membrane; second, the passive efflux of solutes across the sieve tube membrane, dependent on the concentration difference between the sieve tube and the apoplast. The passive loss of solutes is favored by a higher sieve tube solute concentration compared with the surrounding apoplast. The decrease in concentration, as one moves further down the tube, means less water will enter because the water potential difference across the sieve tube membrane decreases, and more so for a more permeable membrane (Figure 2D). Consequently, due to the volume conservation, the increase in axial velocity is less for more permeable membranes (Figure 2C) and there is also a decrease in axial solute flux (Figure 2E). Due to the concentration difference across the sieve tube membrane there is solute efflux which increases for smaller values of the reflection coefficient, σ, corresponding to a leakier membrane (Figure 2F). However, the sieve tube membrane solute permeability, *P*_*s*_, is in the order of 10^−10^ m s^−1^ (see Appendix A), and too small to cause dramatic changes in the sieve tube concentration. If the permeability of the sieve tube membrane, *P*_*s*_, increases (with a corresponding decrease in the reflection coefficient, σ) there is a smaller osmotic effect on water exchange, leading to less water entering the sieve tube (Figure 2D) and a smaller decrease of solute concentration (Figure 2B). Eventually, for a very permeable membrane there is a reversal and water moves out radially for σ < 0.7 (Figure 2D), and the axial velocity decreases along the axis (Figure 2C). Simultaneously, the pressure inside the sieve tube decreases less and less for smaller values of σ (Figure 2A). Axial velocity and axial solute flux at one point become null (Figures 2C,E). Numerical calculation has to stop at this point since, without specifying boundary conditions at the outlet of the tube, it cannot be decided whether there is inflow from the opposite side or the axial velocity remains zero along the tube.

The expected behavior of solute concentration 〈*ĉ*〉 for very permeable membranes (small values of σ) would be that 〈*ĉ*〉 decreases less with distance, approaching a value constant over distance for σ = 0, but not increasing over the initial value at *z* = 0. Obviously our numerical results of solute concentration (Figure 2B) deviate from this expected behavior. The steep increase in 〈*ĉ*〉 (Figure 2B) for very small values of σ stems from the fact that the pressure gradient on the left hand side of Equation (31) approaches zero much faster than radial solute flow on the right hand side. The extreme of this scenario is seen for a totally permeable membrane (σ = 0), in which case the radial water exchange is driven solely by the pressure difference across the sieve tube membrane, and the solute flux occurs predominantly through convection by the water moving across the sieve tube membrane. In this case Equation (31) becomes $\frac{\partial {\hat{p}}_{0}}{\partial \hat{z}}\frac{\partial {\hat{c}}_{0}}{\partial \hat{z}}=\left(-{\hat{u}}_{\text{lat 0}}/2+{\hat{P}}_{s}\right)\left({\hat{c}}_{0}-{\hat{c}}_{\text{out}}\right)$, concentration inside the sieve tube *c*_0_(*z*) should stay constant, and apoplast solute concentration *c*_out_(*z*) would be expected to tend to *c*_0_(*z*) as a consequence of the high permeability of the membrane. However, our model assumes *c*_out_(*z*) to be independent of radial solute flux, with constant apoplastic concentration gradient over distance [model assumptions 8 and 9, see also Equation (10)], leading to a higher concentration difference between inside and outside than expected, and, as a mathematical consequence of Equation (31), an accumulation of solute inside the tube with distance. Thus the results for solute concentration (Figure 2B) and radial solute flow (Figure 2F), though consistent with the model assumptions, are not realistic for small values of σ.

### Comparison of zeroth and first order of expansion

In order to justify the truncation of the series expansion we compare zeroth and first order results. For velocity and pressure Equations (23) to (28) the first two orders structurally only differ by expressions proportional to the Reynolds number Re which is quite small in our case (Re = 8.94 × 10^−5^ for the values reported in Table 1). Thus we expect terms proportional to Re not to contribute significantly for the set of parameters given (Table 1). The remaining terms of first order in Equations (24), (26) and (28) should be of the same order of magnitude as zeroth order, depending on boundary conditions. Together with the fact that the aspect ratio ε is small (ε = 2 × 10^−5^ for the values of Table 1) we conclude that zeroth order strongly dominates the series expansions of velocity and pressure profiles.

**Figure 3:**
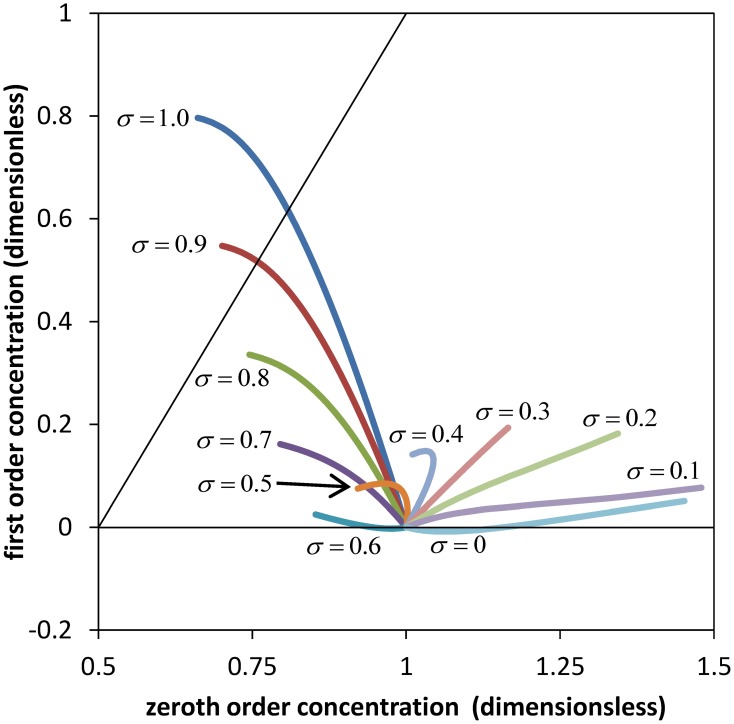
**Comparison of zeroth and first order results**. Average solute concentration for different values of the reflection σ and axial position *ẑ* between *ẑ* = 0 and *ẑ* = *ẑ*_max_ (see Figure 2). All curves start at *ĉ*_0_ (*ẑ* = 0) = 1 and *ĉ*_1_ (*ẑ* = 0) = 0. Parameters of Table 1 were used for the numerical calculation. The diagonal indicates same order of magnitude of zeroth and first order coefficients in the power series expansion.

The comparison is less obvious for the concentration profile where first order Equation (32) is proportional to the Péclet number Pe_*r*_. Since the Péclet numbers in our case are Pe_*r*_ = 3.62 and Pe_*z*_ = 1.81 × 10^5^, it is obvious from Equation (19) that the diffusion component of the solute flux in the axial direction is negligible compared to the convection component. However this situation does not occur for the radial direction, Equation (18). Clearly, diffusive processes (in this case passive) can be important for solute exchange between the sieve tube and its surroundings, and especially if the radial convection flow is small. Figure 3 shows a comparison of zeroth and first order concentration, i.e., the numerical solutions of Equations (31) and (C.8) for the parameter values of Table 1 and different values of the reflection coefficient σ. First order concentration never exceeds the order of magnitude of zeroth order. Together with the small aspect ratio ε it is thus clear that first order in general does not contribute significantly for this set of parameters (Table 1). This implies that out of the set of dimensionless parameters governing the model behavior together with the three boundary condition values $\hat{p}$_i_, $\hat{p}$_out_ (*ẑ*) and *ĉ*_out_ (*ẑ*), there are only three independent significant ones: ${\hat{L}}_{\text{p}}$, *Ĥ* and the reflection coefficient σ. Permeability $\hat{P}$_s_ depends on σ, see Equation (A.2). Aspect ratio ε, Reynolds number Re (i.e., the effect of inertial forces) and Peclét number Pe_*r*_ play a role only when the first order of the series expansion becomes relevant, such as in wider sieve tubes for which ε is bigger than we have considered (Table A1).

### Sensitivity analysis

**Figure 4:**
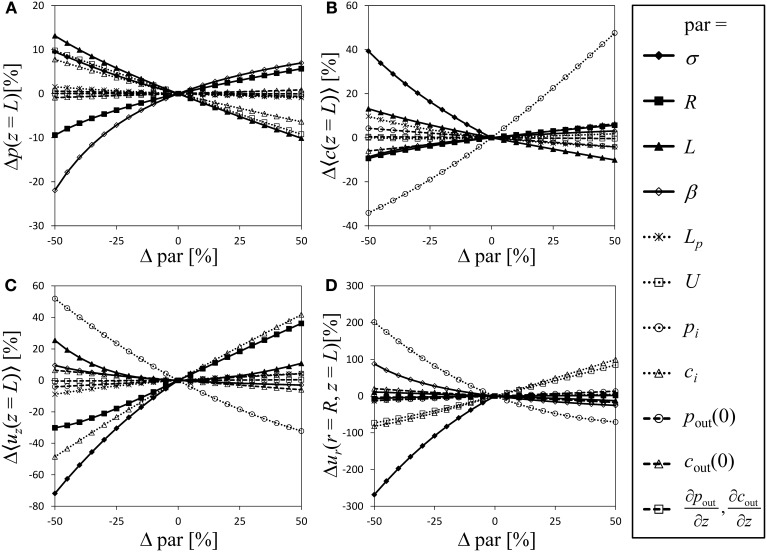
**Sensitivity analysis**. Varying all relevant model parameters up to ±50% from the values of Table 1, the resulting relative change of the state variables (at the end of the tube, i.e., *z* = *L*) is shown as indicator of model sensitivity. State variables are pressure **(A)**, average solute concentration **(B)**, average axial velocity **(C)** and radial velocity at the sieve tube membrane **(D)**. Dimensionless results were converted into original dimensions. Curves are not shown for parameters that give a boundary condition of the corresponding state variable.

For a semipermeable membrane (σ = 1), the exploration of the model behavior for a wide range of possible values of the dimensionless parameters has been provided by Thompson and Holbrook (2003b). Extending this approach to semipermeable membranes would be laborious due to the additional parameters involved. Instead we performed a sensitivity analysis of our model in order to compare the significance of the model parameters, with particular focus on the role the reflection coefficient plays compared to the other parameters, varying each model parameter and boundary condition stepwise from −50 to 50% in relation to the parameter values of Table 1 [which are somewhat similar to the standard parameter set of Thompson and Holbrook (2003b)]. Figure 4 shows the relative change of the state variables pressure, average solute concentration, average axial velocity and radial velocity at the sieve tube membrane. Variation of the parameters causes the dimensionless values of pressure at the inlet of the tube $\hat{p}$(*z* = 0) to differ even if initial pressure *p*_i_ is kept constant. To avoid this effect we converted dimensionless results back into original dimensions for the sensitivity analysis. Since the values of the state variables (except for radial velocity) are fixed by boundary conditions at the inlet of the tube, see Figure 2, changes at the end of the tube (i.e., *z* = *L*) are taken as indicator of model sensitivity. Boundary conditions *p*_i_, *c*_i_, and *U* correspond to state variables *p*, 〈*c*〉, and 〈*u*_*z*_〉, respectively. In cases where these boundary conditions vary, curves of related state variables are not shown in Figure 4 because the variation of initial values dominates the changes of these variables.

Variation of parameters and boundary conditions is straightforward except for the reflection coefficient σ ∈ [0, 1] which is the only parameter limited by a lower as well as an upper boundary. We chose σ = 1 as reference and show negative variation only (such that −50% corresponds to σ = 0.5). Doing so, σ overall appears as one of the most sensitive model parameter, see Figure 4. Given the difficulty of comparing results for varying σ with other (only partially limited) parameters, this result should be treated cautiously regarding a direct numerical comparison of the fractional sensitivities. Still it shows that even small variations of sieve tube solute permeability have a strong effect on the resulting system behavior. Thus there remains no reason to ignore radial solute exchange as it apparently plays in the same league as hydraulic conductivity, geometry and other system properties.

Another obvious result from Figure 4 is that apoplast pressure, concentration and respective gradients (Table 1) do not contribute to the system behavior as strongly as other parameters and boundary conditions. The apoplast surrounding the phloem has been considered to have a constant water potential in most phloem transport models; very few authors (Tyree et al., 1974; Weir, 1981; Thompson and Holbrook, 2003b) considered the more realistic situation of a water potential varying with height. Our result suggests that a water potential gradient can be disregarded, since it constitutes the least influential parameter for all state variables. Nevertheless, in a branched architecture the apoplastic water potential of alternative sinks may well be important (Kaufman and Kramer, 1967; Lang and Düring, 1991).

## Discussion

Solute exchange across sieve tube membranes in the pathway region has hardly been considered in mathematical modeling of phloem transport. Tyree et al. (1974) and more recently Hölttä et al. (2010) and De Schepper and Steppe (2010) have considered a radial solute flow, but in each case the reflection coefficient's value was unity, thus both over-estimating the osmotic contribution to the driving force for solution flow (Equation 8), and decoupling solute and water fluxes (Equation 11). In this study we present a rigorous way of including radial fluxes of both water and solute in the pathway region, in order to give a more realistic analysis of phloem transport. As a first step, we consider passive solute exchange, as a sum of diffusive and convective fluxes across the sieve tube membrane, see Equation (11). Our model considers a diffusive solute flux, described by the irreversible thermodynamics formalism which relates membrane solute permeability, *P*_*s*_, hydraulic conductivity, *L*_p_, and solute reflection coefficient, σ (Appendix A) (Kedem and Katchalsky, 1958).

In the following we compare our approach to the two main papers it is an extension of, i.e., Phillips and Dungan (1993) and Thompson and Holbrook (2003b). In both papers there are just two dimensionless parameters governing the system behavior, ${\hat{L}}_{\text{p}}$ and *Ĥ* [$\hat{R}$ and $\hat{F}$ in the notation of Thompson and Holbrook (2003b)], whereas we have three independent parameters ${\hat{L}}_{\text{p}}$, *Ĥ*, σ when calculating the zeroth order of the series expansion, together with three boundary condition values $\hat{p}$_i_, $\hat{p}$_out_ (*ẑ*) and *ĉ*_out_ (*ẑ*). Aspect ratio ε, Reynolds number Re and Peclét number Pe_*r*_ enter the final equations if calculating the first order of the series expansion, as we did in contrast to Phillips and Dungan (1993). The main difference to Thompson and Holbrook (2003b) (except for allowance of radial solute exchange) is in the boundary conditions: with a permeable membrane, solute flux density is no longer constant along the transport pathway, thus an assumption of a fixed concentration value at the end of the tube becomes numerically intricate when comparing different values of membrane permeability. We therefore chose the boundary conditions of Phillips and Dungan (1993), i.e., fixed values of flux, concentration and pressure at the inlet of the tube. Unlike Phillips and Dungan (1993) and Thompson and Holbrook (2003b) we refrained from exploring the full range of possible values of the dimensionless parameters but focused instead on the effect of different permeabilities with their different rates of radial solute exchange, given a typical and representative set of physiological parameters.

**Figure 5:**
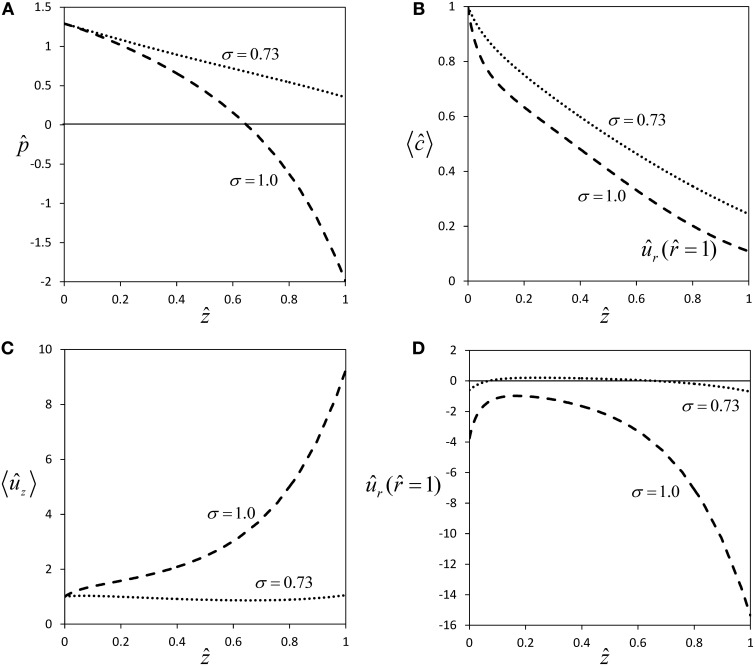
**Effect of membrane solute permeability on flow within long sieve tubes**. Profiles of pressure **(A)**, average concentration **(B)**, average axial velocity **(C)** and radial velocity at sieve tube membrane **(D)** for same parameter set as in Figure 2, except for sieve tube length which was set to *L* = 3 m. The two curves are for semipermeability (σ = 1.0), and for the permeability which gives approximate equilibrium of radial water potential (σ = 0.73).

For a semipermeable membrane (σ = 1), radial water exchange across the sieve tube membrane leads to a strong deviation from Poiseuille flow (Figures 2A,C), the more so the longer the sieve tube. For very long sieve tubes the model predicts that pressure can become negative [Figure 5A, this phenomenon was called “mathematical plasmolysis” by Thompson and Holbrook (2003b)] and an unrealistically steep increase of axial velocity [“runaway phenomenon”, Tyree et al. (1974)] is observed (Figure 5C). These effects are weakened for the more realistic case of a permeable sieve tube membrane (σ < 1), see Figure 2. According to Equation (33), for a certain value of the reflection coefficient, i.e., for σ ≈ 0.73 for our choice of parameter values (Table 1), there is almost no water influx [*û*_*r*_ ($\hat{r}$ = 1) ≈ 0, see Figure 5D], pressure stays positive (Figure 5A) and the “runaway phenomenon” of axial velocity does not occur (Figure 5C). These results show that longer sieve tubes can sustain more stable flow conditions than sieve tubes limited by semipermeable membranes if solute efflux occurs, because of the smaller radial water flux (Figures 2D and 5D) which leads to a less severe dilution of sap (Figures 2B and 5B). At the special value of solute permeability corresponding to σ ≈ 0.73, pressure and axial velocity get closest to Poiseuille flow, and there is (approximate) radial water potential equilibrium as can be seen from Equation (20).

The assumption that sieve tubes are in water potential equilibrium with their surrounding apoplast in plant stems is generally made when measuring turgor pressure gradients indirectly for sieve tubes (Kaufman and Kramer, 1967; Wright and Fisher, 1980; Sovonick-Dunford et al., 1981). Murphy (1989) showed that symplastic connectivity between sieve tubes and the surrounding tissue would not greatly affect the approximation of radial water potential equilibrium. Although our results refer to an idealized sieve tube with no active solute transport, we conclude that this common assumption is more realistic in phloem transport modeling if sieve tubes are taken as permeable, because the value of the water potential difference, ΔΨ, across the sieve tube membrane is approaching zero only for a permeable membrane (Figure 5D), so that ΔΨ ≈ 0 for σ ≈ 0.73. Assuming this *quasi* radial water potential equilibrium, and since ΔΨ = Ψ – Ψ_out_, the sieve tube axial turgor pressure gradient is simply given by the sum of the axial gradients, of the external water potential and the internal osmotic pressure. That is:

(34) $$\frac{dp}{dz}≅\frac{d\Psi_{out}}{dz}+\frac{d\Pi }{dz}$$

Ignoring ΔΨ between sieve tubes and the apoplast, Equation (34) should yield good estimates of phloem turgor pressure and turgor pressure gradients specifically in pathway regions, as in stems.

Clearly, metabolism and storage processes occur throughout the stems of plants, especially in big specimens like trees, and so significant radial solute fluxes must occur (Minchin and Thorpe, 1987; Van Bel, 1990; Hoch et al., 2003). Our value of sieve tube membrane permeability to solutes, *P*_*s*_, (in this case sugars) lies in the bottom range of the values reported for plant cells, 10^−10^ to 10^−6^ m s^−1^, and it is smaller than the values reported for non-charged solutes (Diamond and Wright, 1969; Nobel, 2009). Our choice of parameter values is justified in Appendix A. This small sieve tube membrane permeability to solutes reflects the efficient specialization of sieve tube elements to bulk flow, as it minimizes the diffusive solute loss across the sieve tube membrane. Hence, we have a more efficient system of carbon transport along the plant body that is built to keep solute losses at minimum. Such an efficient transport system is especially important for big specimens where source and sink regions are far apart. For example, in the case of Cucurbitaceae, the combination of a small solute sieve tube membrane permeability to sugars, e.g., sucrose, with the fact that they transport oligosaccharides that exist in the phloem only and seem not to leave it diffusively (Webb and Gorham, 1964, 1965; Schaffer et al., 1996), further illustrates a specialization of sieve tubes to a more efficient system for solute transport bulk flow.

## Outlook

In this study we have applied the Navier–Stokes equation with boundary conditions that describe radial fluxes of both water and solutes to allow a better understanding of phloem transport dynamics. Reloading of solutes through active processes could be added to the boundary condition Equation (11). Whether described by the product of a transport coefficient and the apoplast solute concentration, or by Michaelis–Menten kinetics, the effect of solute reloading on phloem flow would be mainly to counteract the passive solute loss and draw less water into the sieve tube (Figure 2D). Consequently the changes of turgor pressure, velocity and concentration with axial distance would be less than our predictions where there is only solute loss. Also, it would further illustrate the benefit for bulk flow in sieve tubes from minimizing radial solute loss. Thus the changes seen in both the axial velocity and axial solute flux would be attenuated and their profiles would be closer to a more stable system (Figures 2C,F). Additionally, if the reloading of solutes were taken into account, it would be necessary to formulate mechanisms that affect apoplastic concentration in relation to sieve tube solute concentration. This also would help to obtain a more realistic model behavior for very permeable membranes (σ < 0.5). By testing several hypothetical reloading mechanisms (e.g., linear function, Michaelis-Menten) together with possible functions that describe apoplastic concentration over distance, we could gain insight into how plants may have specialized in order to cope with scenarios or situations in which their membrane permeability is affected.

The resistance imposed by sub cellular structures, i.e., cytoplasmic components, phloem proteins (P-protein), and their relation to sieve plates, could be added to the sieve plate impedance factor β, affecting the sieve tube resistance only, and described by the viscous term of the Navier–Stokes equation. The effects observed in our model would thus be enhanced, i.e., the pressure changes due to viscous losses. Hence, the problem turns out to be how to best describe the influence of those sub cellular structures on the overall resistance of the pathway. It would also be informative to investigate the effect of a different sieve tube element structure e.g., where surface arrangement affects the areas for flows in membrane and lumen, as with inclined sieve plates.

For the interpretation of phloem flow measurements, it is of significance that our model shows that, out of all the parameters, a crucial one, the reflection coefficient, typically is the least well known. Its importance suggests that it might be possible to estimate the value of the reflection coefficient by calibrating the model with experimental data, if other relevant parameters are sufficiently well known.

### Conflict of interest statement

The authors declare that the research was conducted in the absence of any commercial or financial relationships that could be construed as a potential conflict of interest.

## Appendix A. physiological parameters

### Sieve tube structure

Sieve elements are elongated with the longitudinal axis parallel to the bundle of vascular tissues. Their length is several to many times greater than their width, but both dimensions can vary considerably within the same plant and between species and genera. Typically, the sieve element length varies from 100 to 5000 μm while its width varies between 10 and 400 μm, for an approximately cylindrical shape (Esau, 1969; Parthasarathy, 1975). Therefore, for a sieve tube length, *L*, of 0.5 m, comparable to a small plant, we specified the following dimensions of the sieve tube elements: radius *R* = l0 μm; length *l* = 250 μm; with 0.5 as the fraction of sieve plate area composed of sieve plate pore area; sieve plate pore radius *r*_*p*_ = 0.23 μm; sieve plate pore length, which equals sieve plate thickness, *l*_*p*_ = 0.5 μm (Thompson and Holbrook, 2003a,b). With these dimensions the sieve plate impedance factor β [Equation (1)] is 0.079, which accounts for the sieve plate contribution to the total sieve tube axial conductivity.

### Sap viscosity

The phloem sap viscosity depends on both temperature and phloem sap chemical composition (proteins, sugars and other solutes). According to Chirife and Buera (1997) the viscosity of sugar solutions at a given temperature T is given by:

(A.1) $$\mu \left(\phi ,\text{T}\right)=\mu_{0}\left(\text{T}\right)a\left(\text{T}\right)\exp\left(E\left(\text{T}\right)x_{s}\right)$$

where μ_0_(*T*) is the viscosity of the solvent, in this case water, at absolute temperature T; *x*_*s*_ is the mole fraction of sugars and *a*(T) and *E*(T) are parameters depending on both temperature and sugar species. For a temperature of 22°C (295.15 K), μ_0_ = 9.548 × 10^−4^ Pa s (Kundu and Cohen, 2008), *a* = 0.905 and *E* = 57.19 (Chirife and Buera, 1997). However, for convenience, as mentioned in the model assumptions we consider phloem sap viscosity as constant (μ = 1.5 mPa s) well inside the range (1–2 mPa s) for typical values of sucrose concentration in sieve tubes, 300–900 mol m^−3^ (Taiz and Zeiger, 1998).

### Turgor pressure

There are not many published results on phloem turgor pressure. Considering both the nature and location of phloem in the plant body, it is clearly difficult not only to get access but also to measure its turgor pressure without damage. The first measurement of turgor pressure in the phloem was by Buttery and Boatman (Buttery and Boatman, 1964) using adapted manometers directly inserted in the laticiferous tissue of Pará rubber tree (*Hevea brasiliensis*). Although not belonging to the translocation pathway, the latex vessel system and sieve tubes are intimately associated elements so that the latex vessel system is considered part of the phloem tissue in *Hevea* and its turgor pressure was assumed to represent values in sieve tubes (Nicole et al., 1991). However, the first measurements of sieve tube turgor pressure (Hammel, 1968) used a manometric device, improved from that of Buttery and Boatman (1964), inserted in the secondary phloem of red Oak trunk (*Quercus rubra*). Hammel (1968) also observed gradients in osmotic pressure, pressure reducing at 0.03 MPa m^−1^ in the direction of flow from source to sink. However, those values were surprisingly low. According to the Münch hypothesis, higher values of turgor and osmotic pressure gradients were expected for the flows and sieve element anatomy of oak. Later, using pressure transducers and the phloem needle technique of Hammel (1968), other species were examined: white ash trunk (*Fraxinus americana* L.) (Sovonick-Dunford et al., 1981; Lee, 1981a,b); Manna ash (*Fraxinus ornus*) (Milburn, 1980); squirting cucumber stems (*Ecballium elaterium*) (Sheikholeslam and Currier, 1977a,b). By using a pressure bomb and Bourdon-type gauge Milburn and Zimmermann (1977) measured pressures in coconut palm inflorescences (*Cocos nucifera* L.). Milburn (1980), using a glass-spiral pressure gauge, measured pressure in castor bean (*Ricinus communis*) sieve tubes. Wright and Fisher (1980) developed another direct way of measuring sieve tubes turgor pressure using severed aphid stylets with attached capillary micromanometers to indicate pressure in sieve tubes of Babylon willow (*Salix babylonica*) stems and in bark strips of sandbar willow (*Salix exigua*) Wright and Fisher (1983). Following Wright and Fisher (1980), Fisher and Cash-Clark (2000) measured sieve tube pressure in the peduncle of wheat. Following the same method, but with an on-line sensor, Gould et al. (2004) presented the second work on a smaller species sow thistle (*Sonchus oleraceus*). In an indirect method, phloem turgor pressure was determined by Kaufman and Kramer (1967) from measurements of tissue water potential along with phloem sap osmotic pressure in red maple (*Acer rubrum* L.), in reasonable agreement with Hammel (1968). Using the same method, Rogers and Peel (1975) observed turgor pressure gradients in stems of osier (*Salix viminalis*), and purple willow (*Salix purpurea*). Based on these findings, for our modeling purposes turgor pressure at entry to the system was set to *p*_i_ = 1 MPa.

### Sieve tube membrane hydraulic conductivity, *L*_*P*_

One of the main parameters describing sieve tube membrane transport is the membrane hydraulic conductivity, *L*_p_. There are not many data on *L*_p_. The first measurements, by Milburn (1974) on castor bean (*Ricinus communis* L.) bark segments, gave values between 5.7 and 8.8 × 10^−14^ m s^−1^ Pa^−1^. Sovonick-Dunford et al. (1982) obtained a value of 9.6 × 10^−15^ m s^−1^ Pa^−1^ for the sieve tube elements of secondary phloem of red oak stem (*Quercus rubra*). Wright and Fisher (1983) measured 5 × 10^−15^ m s^−1^ Pa^−1^ on sandbar willow (*Salix exigua*) bark strips. Before these investigations, most phloem transport models used the hydraulic conductivity published for membranes of other types of plant cells. However, most of that work was for algal cells. Tyree's (1970) review found the membrane hydraulic conductivity for plant cells to range from 5 × 10^−15^ to 1 × 10^−12^ m s^−1^ Pa^−1^. Thus, when compared with other cells, it seems that sieve tubes are at the bottom of that range. In this study, sieve tube membrane hydraulic conductivity, *L*_p_, will be taken as 5 × 10^−15^ m s^−1^ Pa^−1^.

### Solute permeability, P_*S*_, and reflection coefficient, σ

Of all the biological parameters used to describe phloem transport, the reflection coefficient of sieve tube membrane to solutes, σ, and the membrane permeability to those same solutes, *P*_*s*_, are the most difficult to determine. From an irreversible- thermodynamic analysis of solute and water transport across a membrane, Kedem and Katchalsky (1958) showed that the membrane parameters *P*_*s*_ and σ should be related:

(A.2) $$P_{\text{S}}=R_{g}T\frac{L_{\text{p}}}{{\bar{V}}_{\text{S}}}\left(1-\sigma \right)$$

*V*_S_ is the specific volume of the solute. To our knowledge there are no measurements of these parameters for the sieve tube element/companion cell complexes. Hence, one can only speculate from studies of other types of plant cells. Diamond and Wright (1969) presented an empirical relation between the parameters for non-electrolytes from experimental data of *Nitella mucromata* cells. Although they do not present any theoretical justification, the data show reasonably well that σ decreases closely in parallel with increasing permeability *P*_*s*_. The permeability was smaller than 10^−8^ m s^−1^ for σ greater than 0.8, and of the order of 10^−5^ m s^−1^ for σ ≅ 0. From a survey of experimental data on plant cells, Kargol et al. (1997) found a very good agreement with Equation (A.2) in measurements from maize roots, but not with measurements from isolated cells of *Nitella translucens*. Kargol et al. (1997) observed that the linear relationship of Kedem and Katchalsky agrees more with experimental data from artificial membranes. Kargol and Kargol (2000) showed a better agreement with experimental data when the right hand side of Equation (A.2) is multiplied by *K* = 0.021 ± 0.015. Subsequently, Kargol (2001) showed that *K* depends on the solute concentration within the membrane: *K* = *c**V*_S_. However, as a first approach we will adopt the linear relationship (A.2) multiplying the right hand side by *K* = 0.031, which is closer to the average value obtained for the very few plant species presented by Kargol and Kargol (2000), however none of them referring to phloem tissue specifically. Inserting physiological conditions in sieve tubes (Table 1) into Equation (A.2), the solute permeability of the sieve tube membrane is given by:

(A.3) $$P_{\text{S}}=1.83\times 10^{-9}\left(1-\sigma \right)\;(\text{m }s^{-1})$$

Hence *P*_*s*_ varies between 10^−9^ m s^−1^, for totally permeable membrane (σ = 0), and 10^−11^ m s^−1^, for an almost impermeable membrane (σ = 0.99).

### Apoplastic environment

Connor et al. (1977) and Legge (1985) measured vertical water potential gradients in mountain ash (*Eucalytus regnans* F. Muell.), between −0.007 and −0.034 MPa m^−1^, with increasing height. This agrees with the estimated range of −0.01 to −0.03 MPa m^−1^ of the total water potential gradient necessary to drive transpiration stream in trees (Zimmermann and Brown, 1971). Regarding smaller plants, Begg and Turner (1970) measured apoplastic pressure gradients of 0.08 MPa m^−1^ in tobacco. There are not many studies on solute concentrations in the stem apoplast. Minchin and Thorpe (1984) measured the sucrose concentration in the developing stem of bean (*Phaseolus vulgaris* L.) reducing from basal to apical ends with a gradient of 175 mol m^−4^ approximately, corresponding to an osmotic pressure gradient of 0.4 MPa m^−1^. Zimmermann and Brown (1971) observed a reduction in the osmotic pressure with height in white ash (*Fraxinus Americana*) of 0.02 MPa m^−1^ while Connor et al. (1977) observed 0.01 MPa m^−1^ in mountain ash (*Eucalyptus regnans* F. Muell). Hence, taking the estimates of Connor et al. (1977) and Zimmermann and Brown (1971), we specified an apoplastic water potential gradient, $\frac{d\Psi_{\text{out}}}{dz}$, of 0.03 MPa m^−1^ and apoplastic osmotic pressure gradient, $\frac{d\Pi_{\text{out}}}{dz}$, 0.01 MPa m^−1^, in our model. Therefore the apoplastic pressure gradient, $\frac{dp_{\text{out}}}{dz}$, surrounding the sieve tube will be 0.04 MPa m^−1^, taking the direction of flow from apical to basal ends as is convenient for phloem flow.

**Table A1:** **Pressure and pressure gradient measurements on several plant species**.

**References**	**Species**	**Pressure (MPa)**	**Pressure gradient (MPa m^−1^)**	**Method**
Buttery and Boatman (1964)	*Hevea brasiliensis*	0.3–1.1		Adapted manometer
Hammel (1968)	*Quercus rubra*	0.83–3.06	−0.07	Improved method from Buttery and Boatman (1964)
Sovonick-Dunford et al. (1981); Lee (1981a,b); Milburn (1980)	*Fraxinus americana* L., *Fraxinus ornus*	0.69–1.73		Pressure transducers plus method of Hammel (1968)
Sheikholeslam and Currier (1977a,b)	*Ecballium elaterium*	0.15–1.04		Pressure transducers plus method of Hammel (1968)
Milburn and Zimmermann (1977)	*Cocos nucifera* L.	0.12–0.76		Pressure bomb and Bourdon-type gauge
Milburn (1980)	*Ricinus communis*	0.9–1.09		Glass-spiral pressure gauge
Wright and Fisher (1980)	*Salix babylonica*	0.79		Attached capillary micromanometers
Wright and Fisher (1983)	*Salix exigua*	0.63		Attached capillary micromanometers
Fisher and Cash-Clark (2000)	*Triticum aestivum*	2.3		Attached capillary micromanometers
Gould et al. (2004)	*Sonchus oleraceus*	0.4–1.2		Attached capillary micromanometers
Kaufman and Kramer (1967)	*Acer rubrum* L.	0.39–0.56	−0.08 to 0.05	Indirect from water potential measurements
Rogers and Peel (1975)	*Salix viminalis, Salix purpurea*		−0.27 to −0.05	Indirect from water potential measurements

## Appendix B. model based on local application of hagen-poisseuille equation

Here we show that our zeroth order model Equations (27) and (31) can also be obtained using an approach similar to Thompson and Holbrook (2003a), based on conservation equations and a local application of the Hagen-Poiseuille equation together with appropriate boundary conditions. The same assumptions hold as described in Materials and Methods except for absence of a radial coordinate, i.e., the radial flow profile inside the tube is neglected because the solution inside the tube is regarded as well stirred in the radial direction. Also the diffusion of solutes within sieve tube sap is neglected.

### Volume conservation

We start with considering an infinitesimal volume element [*z* − Δ*z, z*] of the tube along the axial direction. The balance between incoming (axial) and outgoing (axial and lateral) fluxes in the infinitesimal volume element is given by *J* (*z* − Δ*z*) = *J* (*z*) + *J*_lat_(*z*). Here *J* denotes axial volume flux [m^3^ s^−1^] which is related to axial flux density *j* [m s^−1^] by *J* = *A j, A* = π*R*^2^ being the cross section through which axial flux occurs. Lateral area of the volume element is *A*_lat_ = 2 π*R*Δ*z* and lateral volume flux is *J*_lat_ = *A*_lat_ *j*_lat_. Thus

(B.1) $$\frac{j\left(z\right)-j\left(z-\Delta z\right)}{\Delta z}=-\frac{2}{R}j_{\text{lat}}\left(z\right)$$

In analogy to Equation (8) lateral flux density across the membrane is given by Starling's Equation (Nobel, 2009)

(B.2) $$j_{\text{lat}}\left(z\right)=L_{\text{P}}\left\{p\left(z\right)-p_{\text{out}}\left(z\right)-\sigma R_{\text{g}}T\left[c\left(z\right)-c_{\text{out}}\left(z\right)\right]\right\}$$

where the van't Hoff relation for dilute solutions has been inserted. Performing the limitΔ*z* → 0, Equation (B.1) becomes

(B.3) $$\frac{\partial j\left(z\right)}{\partial z}=-\frac{2}{R}j_{\text{lat}}\left(z\right)$$

### Convective flow

Convective flow inside the tube is assumed to emanate from a pressure gradient according to a local applicability of the Hagen-Poiseuille equation such that axial flux density is given by

(B.4) $$j\left(z\right)=-\frac{R^{2}}{8\mu^{*}}\frac{\partial p}{\partial z}\left(z\right)$$

with μ^*^ = μ/β as before.

### Solute conservation

In steady state, incoming and outgoing solute fluxes for the small volume element of the tube have to be balanced:

(B.5) $$\begin{array}{l} J\left(z-\Delta z\right)c\left(z-\Delta z\right)-J\left(z\right)c\left(z\right)-J_{\text{lat}}\left(z\right)\bar{c}\left(z\right)(1-\sigma )\\ \;-\;A_{\text{lat}}P_{\text{s}}\Delta c\left(z\right)=0 \end{array}$$

where the first two terms are axial solute flux into and out of the volume element, respectively. The other two terms describe lateral solute flux as sum of convective flux (solute dragged by solvent) and diffusive flux across the membrane. The concentration difference across the membrane has been abbreviated as Δ*c*(*z*) = *c*(*z*) − *c*_out_(*z*) and *c*(*z*) denotes solute concentration in the membrane, a suitably defined function involving inside and outside concentration, e.g.

$$\bar{c}(z)=\frac{c(z)+c_{\text{out}}(z)}{2}.$$

Dividing Equation (B.5) by volume, inserting *j*(*z* − Δ*z*) = *j*(*z*) + 2Δ*zR*^−1^ *j*_lat_ (*z*) from Equation (B.1) and performing the limit Δ*z* → 0, the solute conservation Equation (B.5) becomes (omitting the z dependence in our notation)

(B.6) $$j\frac{\partial c}{\partial z}=\frac{2}{R}j_{\text{lat}}\left(c-(1-\sigma )\bar{c}\right)-\frac{2}{R}P_{\text{s}}\Delta c$$

The system of differential Equations (B.3), (B.4) and (B.6) can be solved if boundary conditions *p*_i_ := *p*(*z* = 0), *U* := *j*(*z* = 0) and *c*_i_ := *c*(*z* = 0) as well as apoplast conditions *p*_out_ (*z*) and *c*_out_ (*z*) are provided.

### Non-dimensionalization

In order to further simplify the equations we apply the same non-dimensionalization as in section Dimensional Analysis. Equations (B.2), (B.3), (B.4) and (B.6) now read

(B.7) $$\text{Lateral membrane flux}:{\hat{j}}_{\text{lat}}={\hat{L}}_{\text{P}}\left\{\hat{p}-{\hat{p}}_{\text{out}}-\sigma \hat{H}\left(\hat{c}-{\hat{c}}_{\text{out}}\right)\text{​​}\right\}$$

(B.8) $$\text{Volume conservation}:\frac{\partial \hat{j}}{\partial \hat{z}}=-{\hat{j}}_{\text{lat}}$$

(B.9) $$\text{Convective flux}:\frac{\partial \hat{p}}{\partial \hat{z}}=-\hat{j}$$

(B.10) $$\text{Solute conservation}:\hat{j}\frac{\partial \hat{c}}{\partial \hat{z}}={\hat{j}}_{\text{lat}}\left(\hat{c}-(1-\sigma )\hat{\bar{c}}\right)-{\hat{P}}_{\text{s}}\left(\hat{c}-{\hat{c}}_{\text{out}}\right)$$

The four parameters remaining are ${\hat{L}}_{\text{p}}$, *Ĥ*, $\hat{P}$_s_ and σ. Boundary conditions are $\hat{j}$(*ẑ* = 0) = 1, *ĉ*(*ẑ* = 0) = 1 and $\hat{p}\left(\hat{z}=0\right)={\hat{p}}_{\text{i}}:=\frac{p_{\text{i}}R^{2}}{8LU\mu^{*}}$. By eliminating flux density from Equations (B.8) to (B.10) the notation can be further compacted, leaving only two differential equations:

(B.11) $$\frac{\partial^{2}\hat{p}}{\partial {\hat{z}}^{2}}={\hat{L}}_{\text{P}}\left[\hat{p}-{\hat{p}}_{\text{out}}-\sigma \hat{H}\left(\hat{c}-{\hat{c}}_{\text{out}}\right)\right]$$

(B.12) $$\begin{array}{l} \frac{\partial \hat{p}}{\partial \hat{z}}\frac{\partial \hat{c}}{\partial \hat{z}}=-{\hat{L}}_{\text{P}}\left[\hat{p}-{\hat{p}}_{\text{out}}-\sigma \hat{H}\left(\hat{c}-{\hat{c}}_{\text{out}}\right)\right]\left(\hat{c}-(1-\sigma )\hat{\bar{c}}\right)\\ \;+\;{\hat{P}}_{\text{s}}\left(\hat{c}-{\hat{c}}_{\text{out}}\right) \end{array}$$

which are identical to Equations (27) and (31).

## Appendix C. preparing model equations for numerical evaluation

In order to numerically evaluate the system of differential equations (27), (28) and (31), they are transformed into a first order system of differential equations in the following way: inserting Equation (27) into Equation (28) leads to

(C.1) $$\begin{array}{l} \frac{\partial^{2}{\hat{p}}_{1}}{\partial {\hat{z}}^{2}}={\hat{L}}_{\text{P}}\left[{\hat{p}}_{1}-\sigma \hat{H}{\hat{c}}_{1}\left(\hat{r}=1\right)\right]\\ \;-\frac{3\text{Re}}{8}\{{\hat{L}}_{\text{P}}\frac{\partial {\hat{p}}_{0}}{\partial \hat{z}}\left[\frac{\partial {\hat{p}}_{0}}{\partial \hat{z}}-\frac{\partial {\hat{p}}_{\text{out}}}{\partial \hat{z}}-\sigma \hat{H}\left(\frac{\partial {\hat{c}}_{0}}{\partial \hat{z}}-\frac{\partial {\hat{c}}_{\text{out}}}{\partial \hat{z}}\right)\right]\\ \;+\;{\left({\hat{u}}_{\text{lat 0}}\right)}^{2}\} \end{array}$$

where first order concentration at the sieve tube membrane follows from Equation (32)

(C.2) $${\hat{c}}_{1}\left(\hat{r}=1\right)=-\frac{3{\text{Pe}}_{r}}{8}\frac{\partial {\hat{p}}_{0}}{\partial \hat{z}}\frac{\partial {\hat{c}}_{0}}{\partial \hat{z}}-\frac{5{\text{Pe}}_{r}}{24}\frac{\partial {\hat{c}}_{0}(z=0)}{\partial \hat{z}}$$

Thus the system of five first order differential equations to be solved is

(C.3) $$\frac{\partial {\hat{p}}_{0}}{\partial \hat{z}}={\hat{p}}_{0}^{1}$$

(C.4) $$\frac{\partial {\hat{p}}_{1}}{\partial \hat{z}}={\hat{p}}_{1}^{1}$$

(C.5) $$\frac{\partial {\hat{c}}_{0}}{\partial \hat{z}}=\left[-{\hat{u}}_{\text{lat 0}}\left({\hat{c}}_{0}-(1-\sigma ){\hat{\bar{c}}}_{0}\right)+{\hat{P}}_{\text{s}}\left({\hat{c}}_{0}-{\hat{c}}_{\text{out}}\right)\right]/{\hat{p}}_{0}^{1}$$

(C.6) $$\frac{\partial {\hat{p}}_{0}^{1}}{\partial \hat{z}}={\hat{u}}_{\text{lat 0}}$$

(C.7) $$\begin{array}{l} \frac{\partial {\hat{p}}_{1}^{1}}{\partial \hat{z}}={\hat{L}}_{\text{P}}\left\{{\hat{p}}_{1}+\sigma \hat{H}{\text{Pe}}_{r}\left[\frac{3}{8}{\hat{p}}_{0}^{1}\frac{\partial {\hat{c}}_{0}}{\partial \hat{z}}+\frac{5}{24}\frac{\partial {\hat{c}}_{0}(z=0)}{\partial \hat{z}}\right]\right\}\\ \;-\frac{3\text{Re}}{8}\left\{{\left({\hat{u}}_{\text{lat 0}}\right)}^{2}+{\hat{L}}_{\text{P}}{\hat{p}}_{0}^{1}\left[{\hat{p}}_{0}^{1}-\frac{\partial {\hat{p}}_{\text{out}}}{\partial \hat{z}}-\sigma \hat{H}\left(\frac{\partial {\hat{c}}_{0}}{\partial \hat{z}}-\frac{\partial {\hat{c}}_{\text{out}}}{\partial \hat{z}}\right)\right]\right\} \end{array}$$

where *û*_lat 0_ (*ẑ*) is given by Equation (27). Respective initial conditions are $\hat{p}$_0_ (0) = $\hat{p}$_i_, $\hat{p}$_1_ (0) = 0, *ĉ*_0_ (0) = 1, $\hat{p}$^1^_0_ (0) = −1 and $\hat{p}$^1^_1_ (0) = −3*Re*/8 *û*_lat 0_ (0).

Inserting Equation (31) into Equation (32), the average of first order concentration over the cross section becomes

(C.8) $$\langle {\hat{c}}_{1}\rangle =\frac{5{\text{Pe}}_{r}}{24}\left\{{\hat{u}}_{\text{lat 0}}\left({\hat{c}}_{0}-(1-\sigma ){\hat{\bar{c}}}_{0}\right)-{\hat{P}}_{\text{s}}\left({\hat{c}}_{0}-{\hat{c}}_{\text{out}}\right)-\frac{\partial {\hat{c}}_{0}(z=0)}{\partial \hat{z}}\right\}$$

The average of axial velocity (zeroth plus first order) over cross section follows from Equations (23) and (24) as

(C.9) $$\langle {\hat{u}}_{z}\rangle =-\frac{\partial {\hat{p}}_{0}}{\partial \hat{z}}-\varepsilon \left(\frac{\partial {\hat{p}}_{1}}{\partial \hat{z}}+\frac{3\text{Re}}{8}\frac{\partial^{2}{\hat{p}}_{0}}{\partial {\hat{z}}^{2}}\frac{\partial {\hat{p}}_{0}}{\partial \hat{z}}\right)$$

Combining Equations (25) and (26) radial velocity (zeroth plus first order) at the sieve tube membrane becomes

(C.10) $${\hat{u}}_{r}\left(\hat{r}=1\right)=\frac{1}{2}\frac{\partial^{2}{\hat{p}}_{0}}{\partial {\hat{z}}^{2}}+\frac{\varepsilon }{2}{\hat{L}}_{\text{P}}\left\{{\hat{p}}_{1}-\sigma \hat{H}{\hat{c}}_{1}\left(\hat{r}=1\right)\right\}$$
